# Impairment of Hyaluronan-Related Glycocalyx Inhibits Macropinocytosis in Cancer Cells via p21 Upregulation

**DOI:** 10.1021/acs.biomac.5c00812

**Published:** 2025-09-03

**Authors:** Guangxin Luan, Qian Guo, Yiwen Liu, Guoliang Zhang, Yan Du, Cuixia Yang, Hui Wang, Jiajie Hu, Yiqing He, Feng Gao

**Affiliations:** 1 Department of Molecular Biology, 667483 Shanghai Sixth People’s Hospital Affiliated to Shanghai Jiao Tong University School of Medicine, Shanghai 200233, China; 2 Department of Clinical Laboratory, Shanghai Sixth People’s Hospital Affiliated to Shanghai Jiao Tong University School of Medicine, Shanghai 200233, China

## Abstract

Macropinocytosis (MP) in cancer cells is an endocytic process for nutrient extraction initiated by membrane ruffling that supports tumor progression. Hyaluronan (HA), a major component of glycocalyx coating the surface of living cells, is reported to influence the membrane morphology. However, whether HA-related glycocalyx (HA-GCX) regulates MP through membrane reconstruction remains unclear. Here, we demonstrated that the HA-GCX thickness was positively correlated with MP activity in cancer cells. Intriguingly, the disruption of HA-GCX could suppress MP by decreasing membrane ruffle formation. Furthermore, the knockdown of cyclin-dependent kinase inhibitor 1 (CDKN1A/p21) and its downstream matrix metalloproteinase (MMP) 1/9, which were identified to be significantly upregulated following HA-GCX removal, could rescue MP. Importantly, the breakdown of HA-GCX could impede cancer cell proliferation through inhibition of MP. Taken together, our study revealed a novel regulatory role of HA-GCX in MP, which might provide a promising therapeutic avenue in cancer treatment.

## Graphical abstract

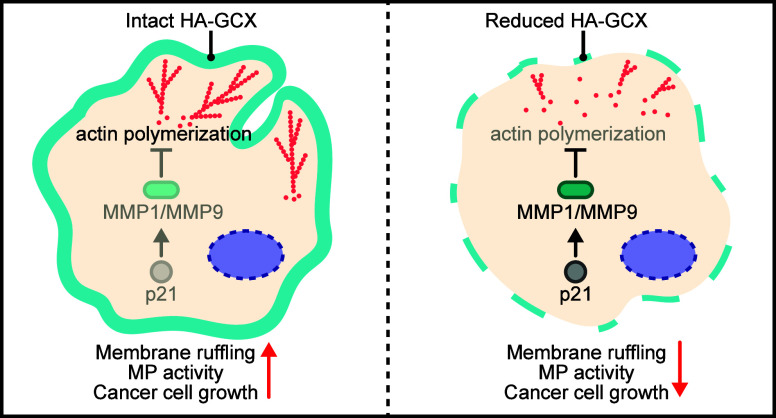

## 1 Introduction

Macropinocytosis (MP) is a highly conserved endocytic process for nonselective internalization of extracellular nutrients in cancer cells and ingestion of pathogens in phagocytes.^1^ MP is characterized by the formation of plasma membrane ruffles and the subsequent generation of large-sized vesicles called macropinosomes.^2^ It is reported that the extracellular matrix (ECM) is the main source of macromolecules for cancer cells to obtain energy under a nutrient-poor environment through MP.^3^ For example, hyaluronan (HA), a major ECM glycosaminoglycan, could be internalized by MP under nutrient deprivation.^4^ Furthermore, studies demonstrate that proline, a key amino acid liberated from collagen degradation, is taken up via MP and serves as a crucial energy source and biosynthetic precursor to enhance cancer cell survival.^5^ During cancer progression, ECM components and construction undergo remodeling both within the tumor stroma and cancer cell membrane including glycocalyx.^6^

Glycocalyx is a gel-like pericellular meshwork coating living cells, which comprises glycoproteins, glycolipids, proteoglycans, and glycosaminoglycans.^7^ In cancer cells, the composition and structure of the glycocalyx changed markedly, leading to an increase in glycocalyx thickness.^8^ It is demonstrated that malignant cancer cells present a denser HA coat, a major component of glycocalyx located at the outer surface of the cell membrane that is implicated in cancer progression such as proliferation, metastasis, and drug resistance.^9^ A previous study has reported that HA polymers in the glycocalyx could regulate the membrane shape, such as the extension of spherical and tubular membrane structures.^10^ Meanwhile, the generation of macropinosomes in MP was found to be dependent on the cellular membrane deformation like ruffles.^11^ Thus, this prompts us to speculate that the HA-related glycocalyx (HA-GCX) might be closely associated with MP activity.

In this study, we aim to investigate the role of HA-GCX impairment in regulating MP and explore the underlying mechanisms. First, we demonstrated a positive correlation between HA-GCX thickness and MP activity in cancer cells. Then, we found that HA-GCX disruption could significantly inhibit MP by impairing actin-dependent membrane ruffle formation, thereby reducing cancer cell proliferation. Furthermore, the removal of HA-GCX robustly upregulated cyclin-dependent kinase inhibitor 1 (CDKN1A/p21) and its downstream targets, matrix metalloproteinase (MMP) 1/9. Notably, the knockdown of p21, MMP1, or MMP9 could restore MP activity, suggesting a critical role in mediating HA-GCX removal-induced MP suppression. Finally, we confirmed that HA-GCX digestion delayed tumor growth through inhibiting p21-MMP1/MMP9 axis-mediated MP *in vivo*. Our findings presented a regulatory mechanism linking HA-GCX remodeling to MP activity that could be exploited to develop therapeutic strategies in cancers.

## 2 Materials and Methods

### 2.1 Cell Culture and Treatment

Human cancer cell lines A549, AGS, BT-549, HT-29, MCF7, MDA-MB-231, MDA-MB-468, MG-63, PANC-1, SW480, and SW620 were purchased from the American Type Culture Collection, and 95-D, HCT116, and SNU-216 were purchased from the National Collection of Authenticated Cell Cultures. All cells were maintained at 37 °C in a humidified incubator with 5% CO_2_ and atmospheric oxygen. 95-D, HT-29, MCF7, MDA-MB-231, MDA-MB-468, MG-63, PANC-1, SW480, and SW620 cells were cultured in Dulbecco’s modified Eagle medium (11995073, Gibco, USA). A549, AGS, and SNU-216 cells were cultured in RPMI medium 1640 (11875093, Gibco, USA). BT-549 cells were cultured in RPMI medium 1640 supplemented with 10 μg/mL insulin. HCT116 cells were cultured in McCoy’s 5A medium (16600082, Gibco, USA). All media were supplemented with 10% fetal bovine serum (A5670701, Gibco, USA) and 1% penicillin/streptomycin (15140122, Gibco, USA).

**Figure 1:**
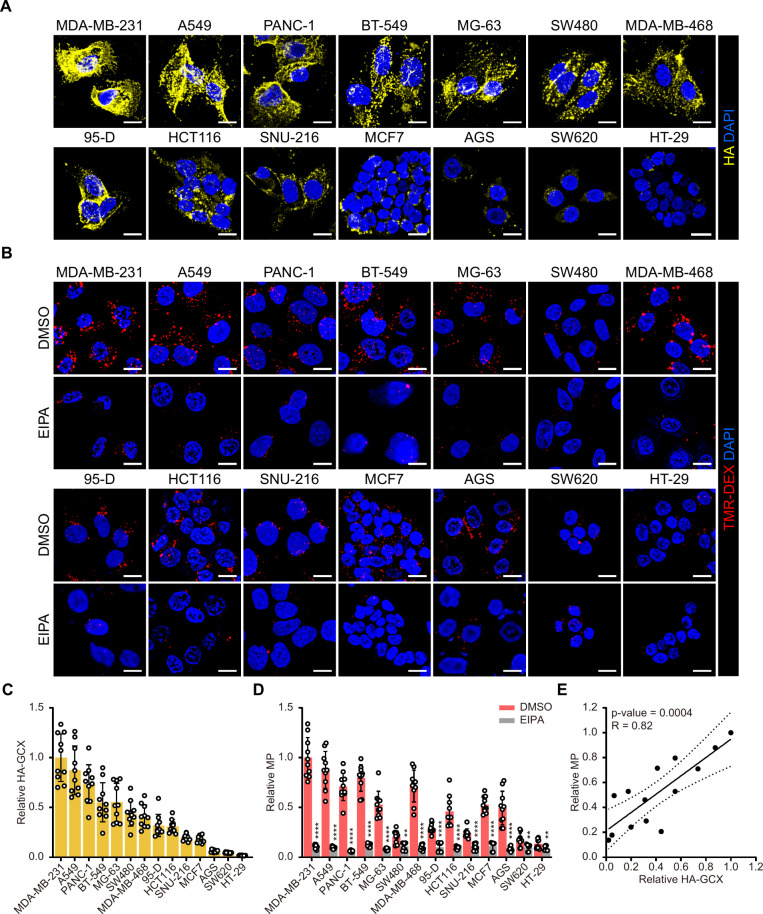
HA-GCX thickness is positively correlated with MP activity in a broad range of cancer cells. (A, B) HA-GCX and MP visualization using b-HABP (yellow) and TMR-DEX (red) in 14 cancer cell lines treated with or without EIPA. Nuclei are stained with DAPI (blue). Scale bars, 10 μm. (C, D) Quantification of relative HA-GCX thickness and MP activity is presented relative to the MDA-MB-231 cell line (*n* = 10). (E) Linear regressions correlating relative HA-GCX thickness with relative MP activity in cancer cell lines (*n* = 14). **p < 0.01, ****p < 0.0001.

Bovine testicular hyaluronidase (HAase; H3506, Sigma-Aldrich, USA) treated cells at a final concentration of 200 U/mL for 24 h (Figures 2, 3, 4A, 5, 6A,B, and 7 and Figures S4 and S5), 200 U/mL for 0–4 days (Figures 4B and 6C,D), or 200 U/mL for 10 days (Figures 4C and 6E,F). This concentration was selected based on a dose–response relationship between HAase and MP activity in MDA-MB-231 cells (Figure S2A), representing the minimum dose required for maximum MP inhibition in our system. Heat-inactivated (HI) HAase was prepared by boiling it for 15 min at 95 °C. HI HAase treated cells at a final concentration of 200 U/mL for 24 h. As a chemical inhibitor of MP,^12^ 5-(*N*-ethyl-*N*-isopropyl) amiloride (EIPA; A3085, Sigma-Aldrich, USA) treated cells at a final concentration of 25 μM for 1.5 h before dextran uptake (Figure 1), 10 μM for 24 h (Figures 2, 4A, and 6A,B and Figure S4), 10 μM for 0–4 days (Figures 4B and 6C,D), or 10 μM for 10 days (Figures 4C and 6E,F). 4-Methylumbelliferone (4-MU; M1381, Sigma-Aldrich, USA) treated cells at a final concentration of 1 mM for 24 h. Cytochalasin D (CytoD; C2618, Sigma-Aldrich, USA) treated cells at a final concentration of 50 nM for 24 h.

**Figure 2:**
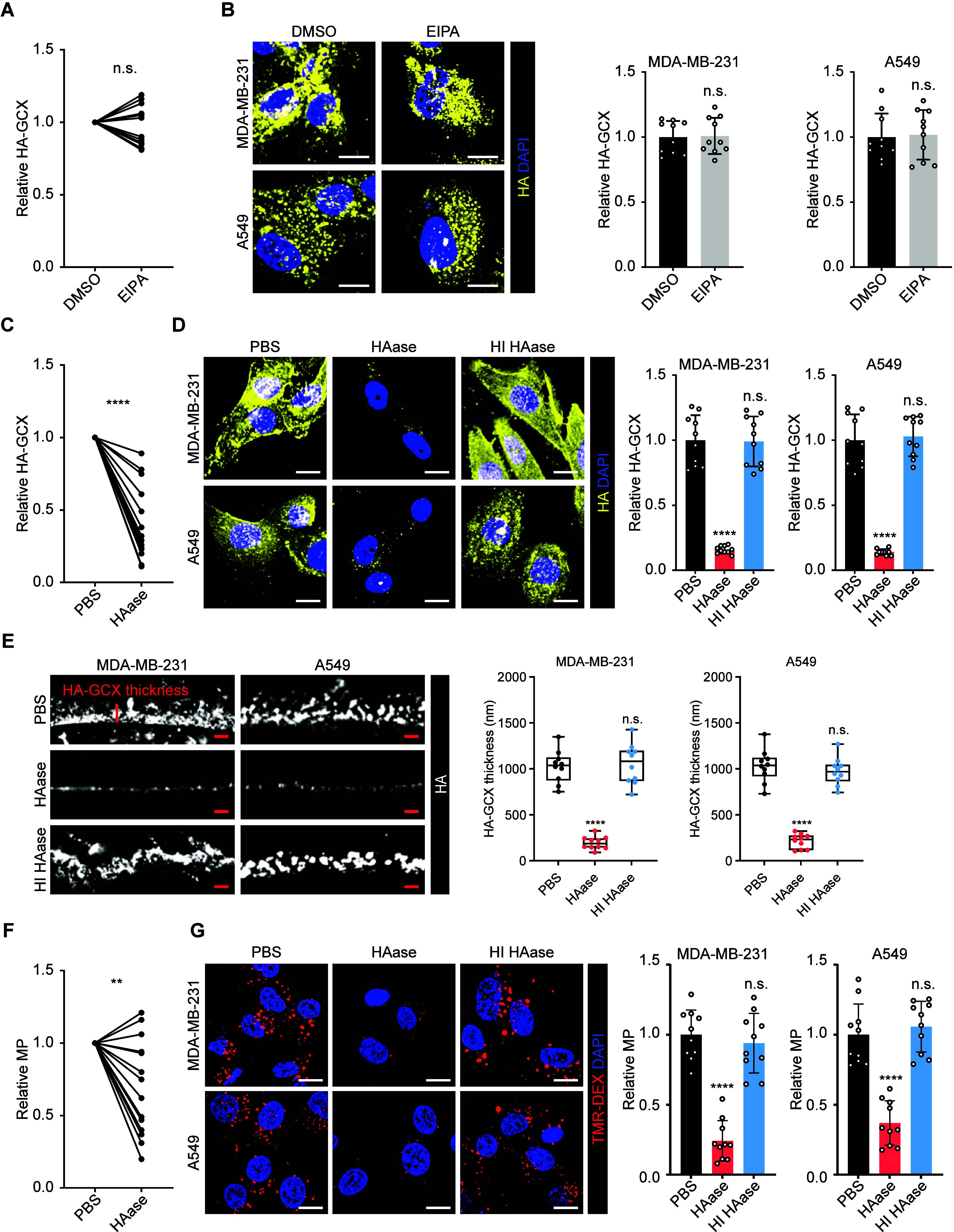
HA-GCX disruption with HAase inhibits MP in cancer cells. (A) Quantification of relative HA-GCX in 14 cancer cell lines treated with or without EIPA is presented relative to the DMSO group (*n* = 14). (B) HA-GCX visualization using b-HABP (yellow) in MDA-MB-231 and A549 cells treated with or without EIPA. Scale bars, 10 μm. Quantification of relative HA-GCX is presented relative to the DMSO group. Mean ± SD (*n* = 10). (C) Quantification of relative HA-GCX in 14 cancer cell lines treated with or without HAase is presented relative to the PBS group (*n* = 14). (D) HA-GCX visualization using b-HABP (yellow) in MDA-MB-231 and A549 cells treated with or without HAase or HI HAase. Scale bars, 10 μm. Quantification of relative HA-GCX is presented relative to the PBS group. Mean ± SD (*n* = 10). (E) Representative 3D-SIM images of HA-GCX (b-HABP, white) in MDA-MB-231 and A549 cells treated with or without HAase or HI HAase. Scale bars, 1 μm. Quantification of HA-GCX thickness (red arrow) is performed at 10 cells (10 points per cell) for each group (*n* = 10 cells). (F) Quantification of relative MP activity in 14 cancer cell lines treated with or without HAase is presented relative to the PBS group (*n* = 14). (G) MP visualization using TMR-DEX (red) in MDA-MB-231 and A549 cells treated with or without HAase or HI HAase. Scale bars, 10 μm. Quantification of relative MP activity is presented relative to the PBS group. Mean ± SD (*n* = 10). **p < 0.01, ****p < 0.0001.

**Figure 3:**
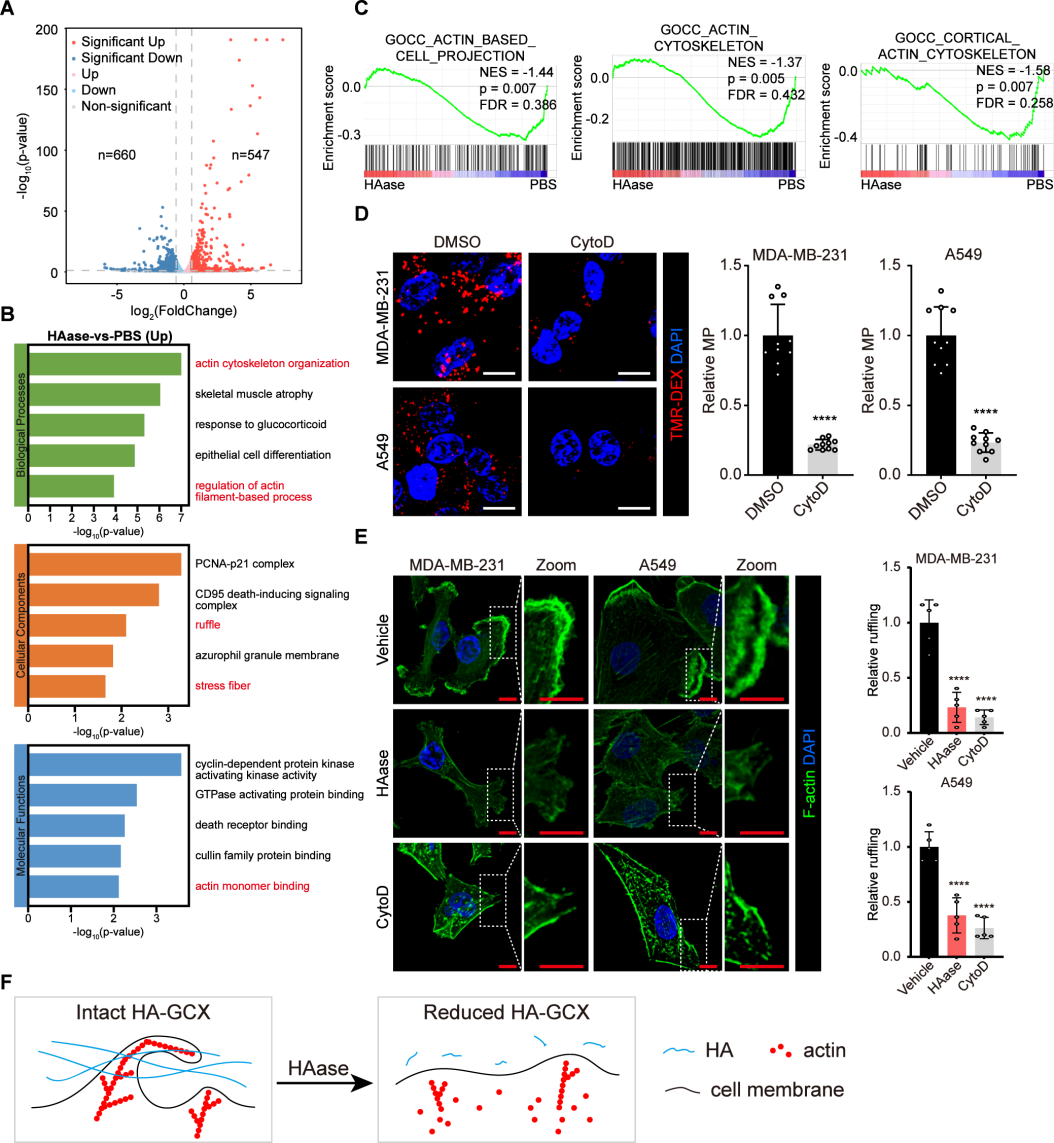
HAase inhibits MP through actin remodeling in cancer cells. (A) Volcano plot of log2 fold changes vs −log10 *p*-value showing transcriptional differences between MDA-MB-231 cells treated with or without HAase. Vertical lines represent the 1.5-fold change cutoff and the horizontal lines indicate the 0.05 *p*-value cutoff. Up- and downregulated genes are highlighted in red and blue, respectively. (B) GO enrichment analysis of upregulated differentially expressed genes between MDA-MB-231 cells treated with or without HAase. (C) GSEA plot for the actin-related signatures in the HAase group compared with the PBS group in MDA-MB-231 cells. FDR, false discovery rate. (D) MP visualization using TMR-DEX (red) in MDA-MB-231 and A549 cells treated with or without CytoD. Scale bars, 10 μm. Quantification of relative MP activity is presented relative to the DMSO group. Mean ± SD (*n* = 10). (E) Representative immunofluorescence staining images of F-actin (phalloidin, green) in MDA-MB-231 and A549 cells treated with or without HAase or CytoD. Scale bars, 10 μm. Quantification of relative membrane-ruffled cells is presented relative to the vehicle group. Mean ± SD (*n* = 5). (F) Schematic representation of the effects of HAase treatment on HA-GCX and actin-dependent membrane ruffle formation. ****p < 0.0001.

**Figure 4:**
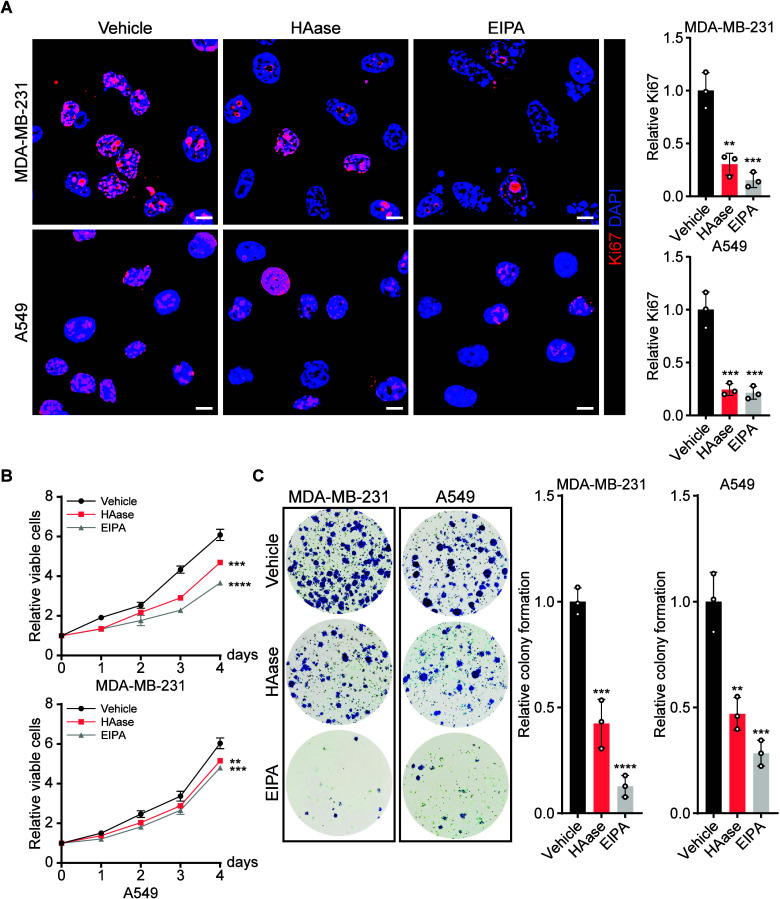
HAase-induced MP suppression reduces cancer cell proliferation. (A) Representative immunofluorescence staining images of Ki67 (red) in MDA-MB-231 and A549 cells treated with or without HAase or EIPA. Scale bars, 10 μm. Quantification of relative Ki67-positive cells is presented relative to the vehicle group. Mean ± SD (*n* = 3). (B) Total viable cells are measured with a CCK-8 assay on the indicated days. Data are presented relative to day 0. Mean ± SD (*n* = 3). (C) Representative clonogenic assay and quantification of the relative colony-forming area. Data are presented relative to the vehicle group. Mean ± SD (*n* = 3). **p < 0.01, ***p < 0.001, and ****p < 0.0001.

**Figure 5:**
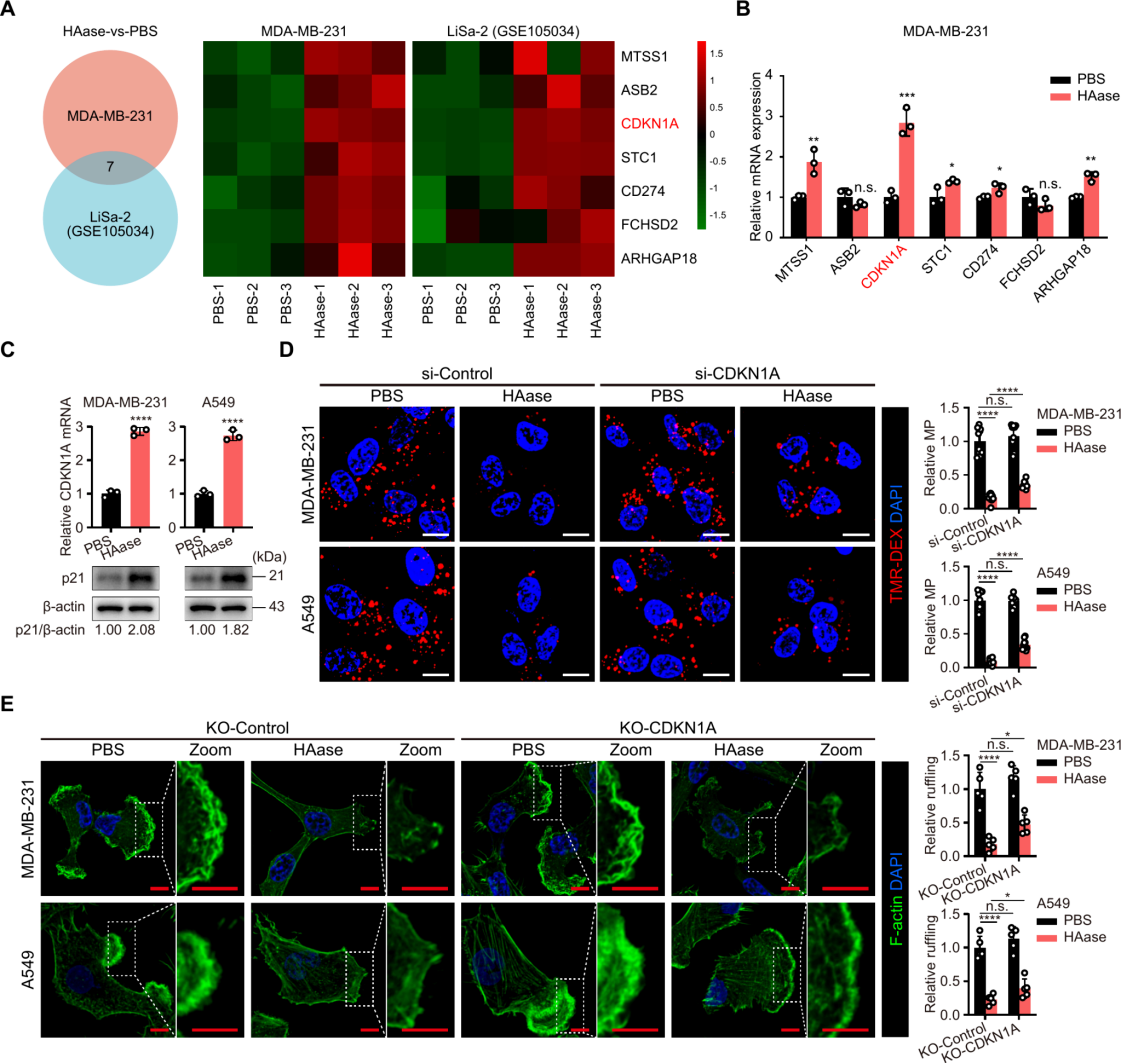
HAase inhibits actin-dependent MP through upregulating p21 in cancer cells. (A) Venn diagram and heat map showing the intersection of the most significantly upregulated actin-related genes between HAase-treated MDA-MB-231 and LiSa-2 (GSE105034) cells. (B) Validation of the expression of candidate genes in MDA-MB-231 cells from the heat map by qPCR. Mean ± SD (*n* = 3). (C) Analysis of CDKN1A/p21 expression by qPCR and Western blotting in MDA-MB-231 and A549 cells treated with or without HAase. Mean ± SD (*n* = 3). (D) MP visualization using TMR-DEX (red) in si-control and si-CDKN1A MDA-MB-231 and A549 cells treated with or without HAase. Scale bars, 10 μm. Quantification of relative MP activity is presented relative to the si-control PBS group. Mean ± SD (*n* = 10). (E) Representative immunofluorescence staining images of F-actin (phalloidin, green) in KO-control and KO-CDKN1A MDA-MB-231 and A549 cells treated with or without HAase. Scale bars, 10 μm. Quantification of relative membrane-ruffled cells is presented relative to the KO-control PBS group. Mean ± SD (*n* = 5). *p < 0.05, **p < 0.01, ***p < 0.001, and ****p < 0.0001.

**Figure 6:**
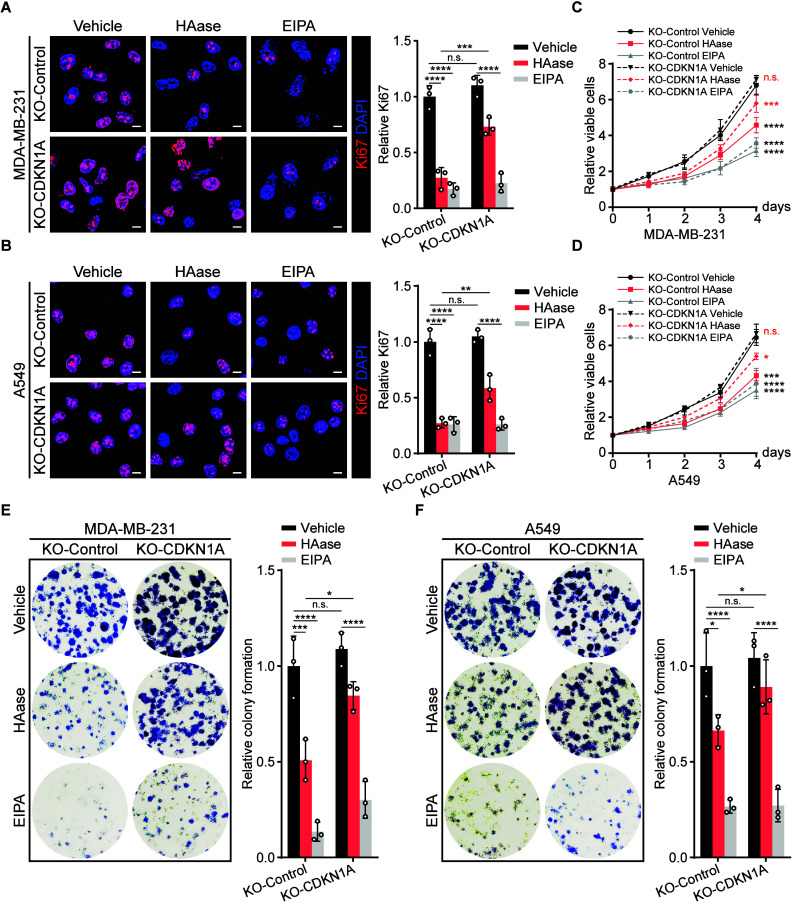
Knockout of CDKN1A reverses the inhibitory effects of HAase on cancer cell proliferation. (A, B) Representative immunofluorescence staining images of Ki67 (red) in KO-control and KO-CDKN1A MDA-MB-231 and A549 cells treated with or without HAase or EIPA. Scale bars, 10 μm. Quantification of relative Ki67-positive cells is presented relative to the KO-control vehicle group. Mean ± SD (*n* = 3). (C, D) Total viable cells are measured with a CCK-8 assay on the indicated days. Data are presented relative to day 0. Mean ± SD (*n* = 3). Black asterisks indicate differences between vehicle and HAase/EIPA groups (same genotype), whereas red asterisks denote differences between KO-control and KO-CDKN1A groups (same treatment). (E, F) Representative clonogenic assay and quantification of the relative colony-forming area. Data are presented relative to the KO-control vehicle group. Mean ± SD (*n* = 3). *p < 0.05, **p < 0.01, ***p < 0.001, and ****p < 0.0001.

**Figure 7:**
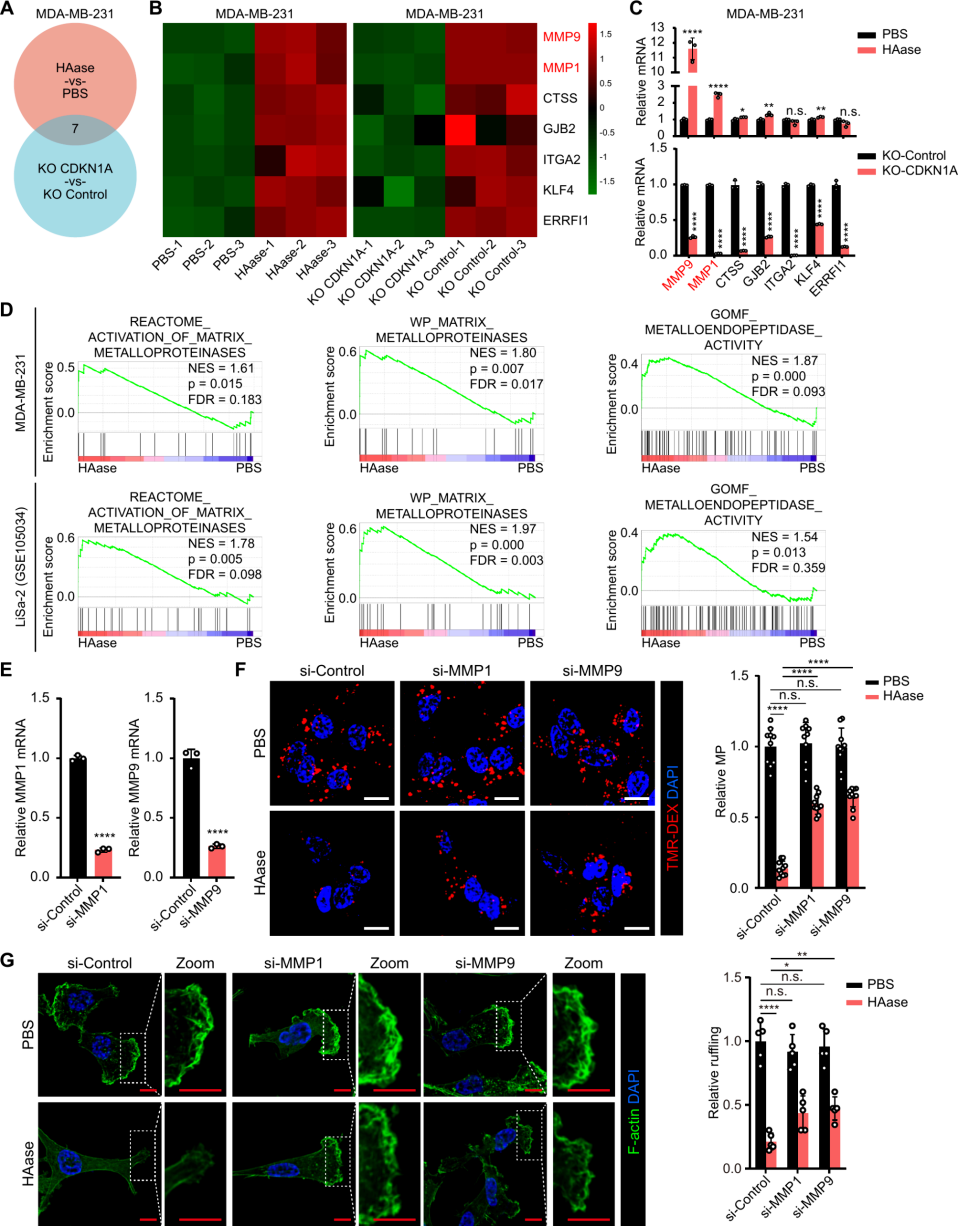
HAase inhibits actin-dependent MP through the p21-MMP1/MMP9 axis in cancer cells. (A, B) Venn diagram and heat map showing the intersection of the most significantly altered genes between HAase-treated and CDKN1A-knockout MDA-MB-231 cells. (C) Validation of the expression of the seven genes from the heat map by qPCR. Mean ± SD (*n* = 3). (D) GSEA plot for the MMP-related signatures in the HAase group compared with the PBS group in MDA-MB-231 and LiSa-2 (GSE105034) cells. FDR, false discovery rate. (E) Analysis of MMP1 and MMP9 knockdown efficiency by qPCR in MDA-MB-231 cells. Mean ± SD (*n* = 3). (F) MP visualization using TMR-DEX (red) in si-control, si-MMP1, and si-MMP9 MDA-MB-231 cells treated with or without HAase. Scale bars, 10 μm. Quantification of relative MP activity is presented relative to the si-control PBS group. Mean ± SD (*n* = 10). (G) Representative immunofluorescence staining images of F-actin (phalloidin, green) in si-control, si-MMP1, and si-MMP9 MDA-MB-231 cells treated with or without HAase. Scale bars, 10 μm. Quantification of relative membrane-ruffled cells is presented relative to the si-control PBS group. Mean ± SD (*n* = 5). *p < 0.05, **p < 0.01, and ****p < 0.0001.

### 2.2 *In Vitro* Macropinocytic Assay

Cells were cultured in 24-well plates with glass bottom for 24 h, followed by serum starvation for 18 h. For macropinosome labeling, cells were incubated with 1 mg/mL tetramethylrhodamine-labeled 70 kDa dextran (TMR-DEX; D1818, Invitrogen, USA) in serum-free medium for 30 min at 37 °C. At the end of the incubation, the cells were rinsed five times with cold PBS and immediately fixed in 4% paraformaldehyde. The macropinocytic uptake index was computed by determining the macropinosome area in relation to the total cell area for each field and then by determining the average across 10 randomly selected fields.^13^

### 2.3 Mouse Xenograft Model

All animal experimental protocols were approved by the Institutional Animal Care and Use Committee at the Shanghai Jiao Tong University School of Medicine, and all procedures were performed in compliance with relevant laws and institutional guidelines. Ten six-week-old female BALB/c nude mice were purchased from the Animal Core of Shanghai Sixth People’s Hospital and randomly divided into two groups: PBS (*n* = 5) and HAase (*n* = 5). Xenografts were generated by injecting 5 × 10^6^ MDA-MB-231 cells suspended in 50% PBS and 50% Matrigel (356234, Corning, USA) into the right mammary fat pads of the mice. The volumes (0.5 × (width^2^ × length)) of the tumors were measured by using digital calipers. When the tumor volume reached 50 mm^3^, mice were administered via direct intratumoral injection of PBS or 1000 U of HAase per tumor every other day before tumor collection.

### 2.4 *Ex Vivo* Macropinocytic Assay

Freshly harvested tumor tissues were cut into pieces of approximately 5 mm cuboidal shape. Tissue fragments were placed in a 24-well plate and injected with 150 μL of a 10 mg/mL TMR-DEX solution. Another 250 μL of a diluted TMR-DEX solution was added to each well to immerse the tissue fragments. The plates were then incubated in the dark for 15 min at room temperature. The tissue fragments were then rinsed twice in PBS before embedding in the O.C.T. compound in a prelabeled cryomold. Specimens were frozen on dry ice and stored at −80 °C for further processing. The macropinocytic uptake index was computed by determining the macropinosome area in relation to the total tumor area for each field and then by determining the average across 10 randomly selected fields.^14^

### 2.5 Immunofluorescence

Cells were seeded in 96-well plates with a glass bottom. The next day, cells were treated as indicated for each experiment and then fixed using 4% paraformaldehyde for 15 min. For biotinylated hyaluronan binding protein (b-HABP) staining, cells were blocked using a streptavidin/biotin blocking kit (SP-2002, Vector Laboratories, USA). For Ki67 staining, cells were permeabilized in 0.1% Triton X-100 for 10 min and then blocked in 5% BSA for 1 h. Cells were probed with b-HABP (1:100; 385911, Sigma-Aldrich, USA) or a Ki67 antibody (1:100; 14-5698-82, Invitrogen, USA) overnight at 4 °C and then incubated with YSFluor 488-conjugated streptavidin (1:1000; 35103ES60, Yeasen, China) or Alexa Fluor 594-conjugated goat antirat IgG (1:1000; ab150160, Abcam, UK) for 1 h at room temperature. To detect actin filaments, cells were stained with iFluor 647-conjugated phalloidin (1:1000; ab176759, Abcam, UK) for 1 h at room temperature. Cell nuclei were stained with 4′,6-diamidino-2-phenylindole (DAPI; 1:1000; D9542, Sigma-Aldrich, USA). Images were captured using a confocal microscope (A1, Nikon, Japan).

The frozen tissue was sliced into 10 μm sections and fixed for 20 min in 4% paraformaldehyde. The following steps were then performed using the same protocol as for cultured cells. Sections were probed with the following primary antibodies: Ki67 (1:100; 14-5698-82, Invitrogen, USA), p21 (1:100; ab109520, Abcam, UK), MMP1 (1:100; ab137332, Abcam, UK), and MMP9 (1:100; ab76003, Abcam, UK). The sections were probed with b-HABP (1:100). The primary antibodies and b-HABP were incubated with secondary antibodies and streptavidin, respectively. Images were captured by using a confocal microscope.

### 2.6 Three-Dimensional Structured Illumination Microscopy (3D-SIM)

HA-GCX of indicated cells was stained with b-HABP (1:100) overnight at 4 °C. The cells were then incubated with Alexa Fluor 488-conjugated streptavidin (1:1000) at room temperature for 1 h. A 3D-SIM microscope (A1, Nikon, Japan) was used to capture images with a 100× oil-immersion objective. HA-GCX thickness was measured in 10 cells per group, with the average value calculated from 10 points per cell. Pseudopodia were excluded from the measurements for their undistinguished glycocalyx-cytosol boundary.^15^

### 2.7 Cell Viability Assay

Cell viability was determined using a Cell Counting Kit-8 (CCK-8) assay kit (CK04, Dojindo, Japan). The cells were plated in 96-well plates at a density of 3000 cells/well and incubated overnight prior to treatment. PBS, HAase, or EIPA was added to the wells for 0, 24, 48, 72, or 96 h. Media were replaced every 24 h. Next, 10 μL of the CCK-8 reagent was added to each well. The plates were then incubated at 37 °C for 2 h. The optical density was measured at 450 nm using a microplate reader (Synergy4, BioTek, USA) at days 0, 1, 2, 3, and 4.

### 2.8 Colony Formation Assay

Cells were seeded in 6-well plates at a density of 500 cells/well and incubated overnight prior to treatment. PBS, HAase, or EIPA was added to the wells for 10 days. Media were replaced every 48 h. Next, cells were washed with PBS, fixed with 4% paraformaldehyde for 15 min, stained with crystal violet for 20 min at 37 °C, and rinsed with purified water three times. The stained colonies were then imaged and counted.

### 2.9 RNA Extraction, Reverse Transcription, and Real-Time Quantitative PCR

RNA extraction was conducted using an RNAiso Plus Reagent (9108, Takara, Japan) according to the manufacturer’s instructions. The RNA concentration was quantified using a NanoDrop system (NanoDrop 2000c, Thermo Fisher Scientific, USA). Next, 1 μg of purified RNA was reverse-transcribed into cDNA using a PrimeScript RT reagent kit (RR047Q, Takara, Japan). Real-time PCR experiments were performed using SYBR Green Mix (RR820A, Takara, Japan) according to the manufacturer’s protocol. All values of genes were normalized against those of ACTB, and the relative expression of genes was calculated by using the 2^–ΔΔCt^ method. The primer sequences used for qPCR are listed in Table S1.

### 2.10 Protein Extraction and Western Blotting

Cells were harvested and lysed in RIPA buffer supplemented with a complete protease inhibitor cocktail. Proteins were resolved on sodium dodecyl sulfate-polyacrylamide gels and transferred to a polyvinylidene difluoride membrane. After blocking with 5% fat-free milk, the membrane was stained with the following primary antibodies: anti-p21 (1:1000; ab109199, Abcam, UK) and antibeta actin (1:1000; ab8226, Abcam, UK), followed by incubation with the appropriate secondary HRP-conjugated antibodies. The blots were developed by using a chemiluminescence substrate (34577, Thermo Fisher Scientific, USA). The bands were captured using an imaging system (Amersham Imager 680, GE Healthcare, USA), and the intensity was quantified using ImageJ software.

### 2.11 RNA Sequencing and Data Analysis

RNA sequencing analysis was performed by OE Biotech (China) using an Illumina NovaSeq 6000 instrument (Illumina, USA). Fragments per kb per million reads of each gene were calculated using Cufflinks, and read counts were obtained by HTSeq-count. The DESeq (2012) R package was used to perform differential expression analysis. A fold change >1.5 with a *p*-value <0.05 was defined as the threshold for significant differential expression. Gene ontology (GO) enrichment analysis of differentially expressed genes was performed using R based on hypergeometric distribution. Gene set enrichment analysis (GSEA) was conducted to determine whether a set of genes showed statistically significant differences.

### 2.12 siRNA Transfection

siRNA constructs targeting human CDKN1A, MMP1, and MMP9 expression were designed and synthesized by RiboBio (China). Transfection was conducted using a riboFECTTM reagent (C11062, RiboBio, China) according to the manufacturer’s protocol. The siRNA sequences are listed in Table S1.

### 2.13 CRISPR/Cas9-Mediated CDKN1A-Knockout

GeneChem (China) designed and cloned the corresponding sgRNAs into GV392 plasmids containing puromycin resistance and hSpCas9 genes. The sgRNA sequences are listed in Table S1. Then, the edited vectors were packaged into lentiviruses in HEK293T cells. To establish cell lines with stable gene deletion, cells were seeded into 6-well plates at a concentration of 1 × 10^5^ cells per well. After 24 h, cells were infected with lentiviruses (multiplicity of infection = 10) and a Hitrans G P reagent (REVG005, GeneChem, China) according to the manufacturer’s protocol. After 72 h of infection, cells were propagated in a selection medium containing 2 μg/mL puromycin (ant-pr-1, InvivoGen, France) for 48 h.

### 2.14 CARMIL1 and HAS2 Overexpression

The lentiviral overexpression construct for human CARMIL1 was generated by GeneChem. Lentivirus infection was conducted according to the manufacturer’s instructions. After 72 h of infection, cells were propagated in a selection medium containing 2 μg/mL puromycin for 48 h. The HAS2-overexpressed MCF7 cell line was previously established.^16^

### 2.15 Statistical Analysis

Statistical analysis was performed using GraphPad Prism version 10.4.0. In box plots, the center line is the median, the box is delimited by the 25th and 75th percentiles, and whiskers represent the minimum and maximum values. The correlation scatterplot between HA-GCX and MP was analyzed by linear regression. Bar graphs present the mean ± SD. Statistical analysis was performed with unpaired Student’s *t*-test for two-group comparison and one-way analysis of variance for multigroup comparisons. A *p*-value of less than 0.05 was considered statistically significant (*p < 0.05, **p < 0.01, ***p < 0.001, and ****p < 0.0001).

## 3 Results

### 3.1 HA-GCX Thickness Is Positively Correlated with MP Activity in Cancer Cells

Although HA-GCX remodeling and MP are both commonly associated with malignant tumor characteristics,^17,18^ the relationship between these processes remains unclear. To investigate this, we analyzed data from The Cancer Genome Atlas (TCGA) and found a positive correlation between HA synthesis-related genes and MP-related genes across 33 types of human cancers (Figure S1).^19–27^ Then, we assessed HA-GCX and MP activity across a panel of 14 cancer cell lines (Figure 1A–D). The results showed that cancer cell lines with denser HA-GCX, such as MDA-MB-231, A549, and PANC-1, exhibited elevated MP activities. In contrast, cancer cells with lower HA-GCX levels showed reduced MP activities. To further confirm the correlation involved, we used linear regressions to analyze the data and found a positive correlation between HA-GCX thickness and MP activity (Figure 1E).

### 3.2 The HA-GCX Disruption Decreases MP Activity in Cancer Cells

As shown above, the thickness of the HA-GCX is positively related to MP activity. It is reported that HA-GCX could regulate membrane morphology,^10^ which is closely associated with formation of membrane ruffles in MP.^11^ Since MDA-MB-231 and A549 cells exhibited both bulkier HA-GCX and stronger MP activity than other cell lines (Figure 1), we selected these two cell lines to further explore the effects of HA-GCX on MP. As shown in Figure 2A,B, the inhibition of MP by EIPA had no significant effects on HA-GCX among 14 cancer cell lines. Then, we degraded the HA-GCX coating cancer cells by HAase treatment and found that the HA-GCX thickness was significantly reduced, while no effects were observed using HI HAase (Figure 2C–E). Intriguingly, our results showed that the disruption of HA-GCX could robustly inhibit MP activity, while HI HAase treatment had no effect on MP (Figure 2F,G and Figure S2). In addition, the removal of HA-GCX by 4-MU, a chemical inhibitor of endogenous HA synthesis,^28^ could also inhibit MP activity (Figure S3). Collectively, these findings suggested that HA-GCX disruption could decrease MP activity.

### 3.3 The HA-GCX Rupture Inhibits MP through Actin-Driven Membrane Ruffling

To further explore the regulatory mechanism of HA-GCX on MP, we performed RNA sequencing to compare the transcriptomic profiles of MDA-MB-231 cells treated with either PBS or HAase for 24 h. Differential expression analysis identified 547 upregulated and 660 downregulated genes in HA-GCX-impaired cells (fold change >1.5, *p* < 0.05; Figure 3A). In addition, GO enrichment analysis revealed a significant enrichment of actin cytoskeleton-related terms after HAase treatment (Figure 3B), which was further confirmed by GSEA (Figure 3C). These findings suggested that the HA-GCX disruption might reduce the level of actin cytoskeleton remodeling, which was associated with membrane ruffling. Thus, we focused on the role of HA-GCX breakdown in the formation of actin-dependent membrane ruffles. Our results showed that CytoD, a well-established inhibitor of actin polymerization,^29^ could inhibit MP activity in MDA-MB-231 and A549 cells, suggesting the involvement of the actin cytoskeleton in regulating MP (Figure 3D). Furthermore, we found that untreated MDA-MB-231 and A549 cells exhibited high levels of actin-related membrane ruffles, which was consistent with their elevated MP activities. Notably, the impairment of HA-GCX could inhibit the formation of actin-dependent membrane ruffles, which was similar to the effect of CytoD treatment (Figure 3E,F). Taken together, our findings revealed the critical role of HA-GCX in regulating MP through actin-driven membrane ruffling in cancer cells.

### 3.4 HA-GCX-Dependent MP Regulates Cancer Cell Proliferation

Given that MP is closely associated with cancer cell growth,^30^ we next investigated whether the removal of HA-GCX could regulate cancer cell proliferation through MP. Our results showed that Ki67, a widely used marker of cell proliferation,^31^ was significantly reduced after HA-GCX disruption, similar to the decrease observed with EIPA treatment (Figure 4A). Furthermore, we performed rescue experiments by overexpressing CARMIL1, which is a critical mediator of MP that does not influence cell proliferation.^32,33^ As shown in Figure S4A,B, CARMIL1 overexpression restored MP activity and increased Ki67-positive cells in HAase-treated cells. Conversely, pharmacological inhibition of MP activity attenuated the proproliferative effects induced by HA-GCX thickening (Figure S4C–E). The above results were further confirmed by CCK-8 and colony formation assays (Figure 4B,C). Collectively, these findings indicated that HA-GCX disruption-induced MP suppression could inhibit cancer cell proliferation.

### 3.5 Identification of p21 as a Regulator in HA-GCX Disruption-Induced MP Inhibition

As shown above, a significant enrichment of actin cytoskeleton-related genes was responsible for HA-GCX regulated MP. We next explored the molecular mechanisms underlying HA-GCX disruption-reduced actin cytoskeleton reorganization by analyzing our transcriptomic data and the Gene Expression Omnibus data (GSE105034). The results showed that seven genes were significantly upregulated in both HAase-treated MDA-MB-231 and LiSa-2 cells, among which p21 showed the most robust increase (Figure 5A–C).

To further investigate the role of p21 in MP activity, we knocked down p21 in MDA-MB-231 and A549 cells. The results showed that the downregulation of p21 could attenuate HA-GCX removal-induced reduction in MP activity (Figure 5D and Figure S5A), suggesting that HA-GCX disruption might inhibit MP by upregulating p21 expression. Meanwhile, the knockout of p21 could recover the membrane ruffle formation (Figure 5E and Figure S5B), implying the negative regulatory role of p21 in actin-dependent membrane ruffling. Furthermore, our results demonstrated that p21-knockout could restore HA-GCX impairment-induced suppression of cancer cell proliferation (Figure 6). Collectively, these findings identified p21 as a critical mediator of HA-GCX-dependent MP and subsequent cancer cell growth.

### 3.6 The HA-GCX Impairment Inhibits Actin-Dependent MP through the p21-MMP1/MMP9 Axis

To investigate the downstream targets of p21 in HA-GCX disruption-induced MP suppression, we performed an intersectional analysis of differentially expressed genes between the HAase-vs-PBS group and the KO-CDKN1A-vs-KO-control group in MDA-MB-231 cells. The results revealed that seven genes were significantly upregulated in HAase-treated cells but downregulated in p21-knockout cells, among which MMP1 and MMP9 showed the most robust changes (Figure 7A–C). Moreover, the GSEA results confirmed the enrichment of MMP-related gene sets in HAase-treated cells (Figure 7D). To further explore the role of MMP1 and MMP9 in HA-GCX-mediated MP, we knocked down MMP1 and MMP9 in MDA-MB-231 cells (Figure 7E). As shown in Figure 7F,G, the downregulation of MMP1 and MMP9 could restore MP activity and membrane ruffle formation. Taken together, these results suggested that HA-GCX disruption could inhibit actin-dependent MP through the p21-MMP1/MMP9 axis.

### 3.7 The HA-GCX Depletion Inhibits Tumor Growth through Decreased MP *In Vivo*

Encouraged by the *in vitro* results, we next investigated the effect of HA-GCX depletion on tumor growth by employing a mouse xenograft model of breast cancer. As shown in Figure 8A, HAase treatment could significantly reduce both the tumor volume and weight. Immunofluorescence staining for Ki67 further confirmed a decrease in tumor cell proliferation induced by HAase (Figure 8B). Moreover, HAase-treated tumors exhibited decreased HA-GCX and MP activity (Figure 8C,D). These findings indicated that HA-GCX disruption could inhibit tumor growth through impaired MP *in vivo*, which was consistent with our *in vitro* observations. Furthermore, immunofluorescence analysis showed that HAase-treated tumors displayed upregulation of p21, MMP1, and MMP9 expression (Figure 8E–G). Overall, these results confirmed that HA-GCX disruption may suppress tumor growth by reducing p21-MMP1/MMP9 axis-mediated MP *in vivo.*

**Figure 8:**
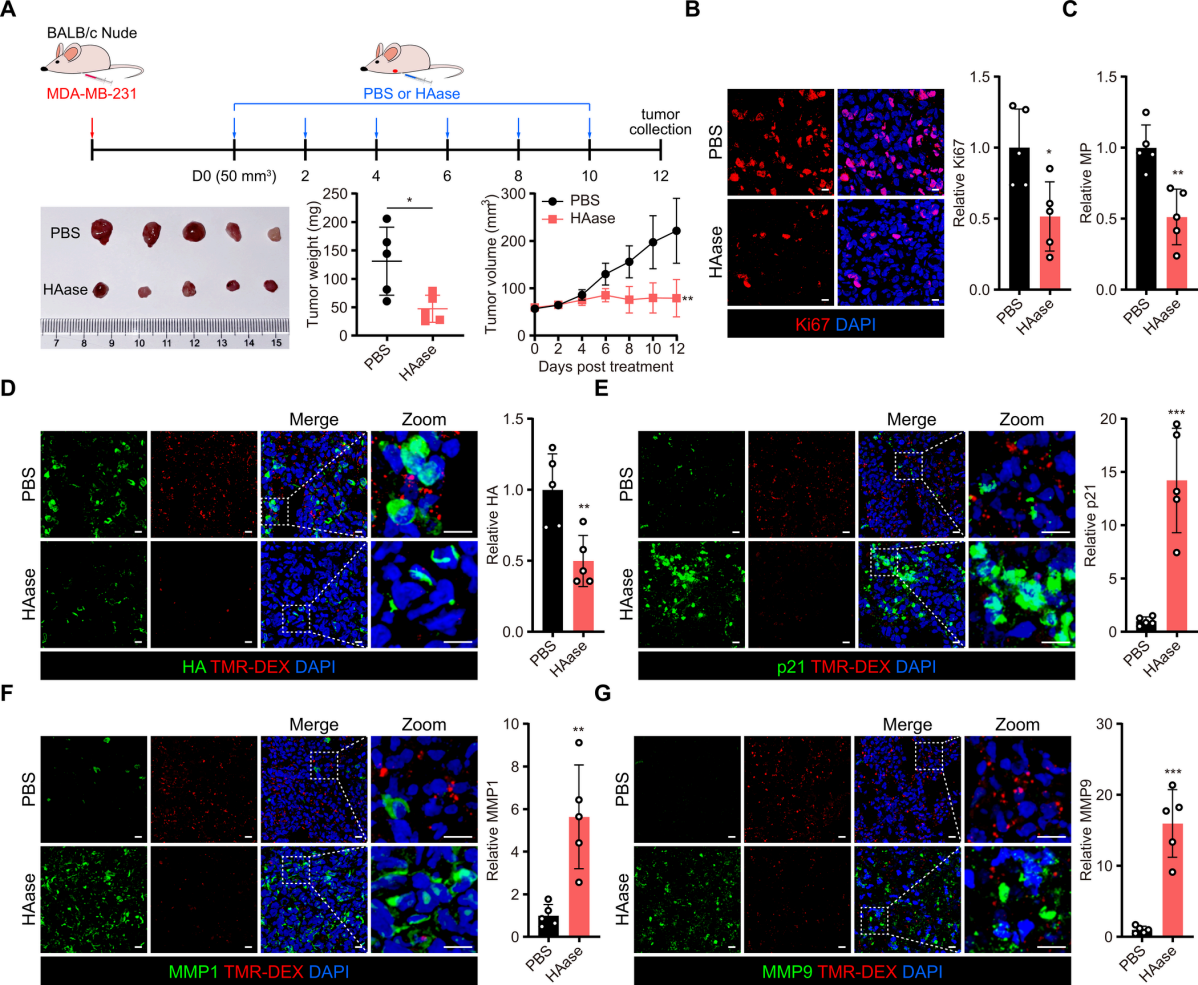
HA-GCX disruption with HAase delays tumor growth by inhibiting MP *in vivo*. (A) Top: six-week-old female BALB/c nude mice are implanted with MDA-MB-231 cells followed by PBS or HAase treatment for 12 days after the tumor volume reached 50 mm^3^. Bottom left: image of collective tumors. Bottom middle: quantification of tumor weight. Mean ± SD (*n* = 5 mice). Bottom right: quantification of the tumor volume on the indicated days after PBS or HAase treatment. Mean ± SD (*n* = 5 mice). (B) Representative immunofluorescence staining images of Ki67 (red) and quantification of relative Ki67-positive cells in MDA-MB-231 xenografts treated with or without HAase. Scale bars, 10 μm. Mean ± SD (*n* = 5 mice). (C–G) Representative immunofluorescence staining images and quantification of relative MP (TMR-DEX), HA-GCX (b-HABP), p21, MMP1, and MMP9 in MDA-MB-231 xenografts treated with or without HAase. Data are presented relative to the PBS group. Scale bars, 10 μm. Mean ± SD (*n* = 5 mice). *p < 0.05, **p < 0.01, and ***p < 0.001.

## 4 Discussion

The extracellular matrix (ECM) is an essential structural scaffold and constitutes a dynamic microenvironment present in all tissues.^34^ In a tumor microenvironment, the remodeling and deposition of ECM components are dysregulated that contribute to cancer progression,^35^ including promoting macropinocytosis (MP) activity. For example, the matrix metalloprotease (MMP)-cleaved type I collagen fragment could promote pancreatic cancer progression by facilitating MP activity.^36^ Glycocalyx, a specific ECM component, is a multifunctional layer of glycans that coats the cell surface.^7^ It is reported that malignant cancer cells exhibit denser glycocalyx to evade immune surveillance and promote tumor progression.^9^ However, few studies have investigated the contribution of glycocalyx to the MP activity. A study has demonstrated that hyaluronan (HA), a major component of glycocalyx, could influence the membrane shape.^10^ As MP is closely related to the generation of membrane ruffling,^37^ we hypothesized that HA-related glycocalyx (HA-GCX) might regulate MP through eliciting membrane reconstruction. In the present study, we found that the HA-GCX thickness was positively correlated to MP activity in a broad range of cancer cell lines. Intriguingly, the removal of HA-GCX could suppress MP activity, leading to inhibition of cancer cell growth. A prior study has reported that a bulky glycocalyx could promote cancer cell growth and survival by facilitating integrin-dependent signaling.^38^ In this study, we uncovered the role of HA-GCX disruption in suppressing cancer cell proliferation through regulating MP activity.

To search for the mechanisms underlying HA-GCX impairment-induced MP inhibition, we further performed a transcriptional sequencing analysis. Our results revealed a significant enrichment of actin-related genes after HA-GCX depletion. As the actin membrane ruffling is a prerequisite for macropinosome formation,^39^ we next sought to validate the contribution of actin cytoskeleton remodeling to HA-GCX-mediated MP activity. Our data showed that the inhibition of actin polymerization could significantly inhibit the formation of actin-driven membrane ruffles, leading to MP downregulation. In accordance, the disruption of HA-GCX could contribute to the same effects in this regard, suggesting that HA-GCX might regulate MP through impairing actin reorganization. A previous study has indicated that mucin-related glycocalyx can modulate actin-dependent membrane deformation, featured as tubule-like projection.^10^ Consistently, our findings showed that HA-GCX could regulate the sheet-like membrane ruffle formation.

We next sought to explore the downstream mediators of HA-GCX rupture-reduced actin reorganization and identified that cyclin-dependent kinase inhibitor 1 (CDKN1A/p21) was significantly upregulated after the disruption of the HA-GCX. Although p21 is well-recognized as a cell cycle regulator,^40^ emerging evidence suggests its involvement in cytoskeletal dynamics. For example, cytoplasmic p21 binds and inhibits ROCK1, resulting in decreased actin polymerization.^41,42^ In this study, we demonstrated that p21 was responsible for cytoskeleton-dependent regulation of MP activity induced by HA-GCX remodeling. However, it is worth noting that the knockdown of p21 only partially rescued the MP process, suggesting the involvement of additional regulatory factors. Given that actin-associated genes MTSS1 and ARHGAP18 were also significantly upregulated following HA-GCX disruption,^43,44^ we speculated that these genes might contribute to the regulation of MP, warranting further exploration. We next identified MMP1 and MMP9 as downstream targets of p21. While previous studies have reported that MMPs promote actin cytoskeleton remodeling,^45,46^ our findings demonstrated that HA-GCX disruption-induced upregulation of MMP1 and MMP9 expression could lead to the suppression of actin-dependent membrane ruffles. This discrepancy may help to explain the dual role of MMPs in either promoting or inhibiting tumor cell migration.^47,48^ Nevertheless, the detailed mechanisms by which extracellular HA-GCX triggers intracellular signal transduction to regulate the p21-MMP1/MMP9 axis remain unknown. Indeed, some studies have reported that HA interacts with its major receptors including CD44 to activate intracellular signaling cascades such as the RAS/MAPK and PI3K/AKT pathways,^49,50^ which are known to regulate p21 transcription.^51–53^ We therefore propose that CD44 may serve as a critical sensor of HA-GCX integrity, thus transducing extracellular cues into intracellular signaling, which needs further investigation.

Encouraged by the *in vitro* results, we further evaluated the contributions of HA-GCX to MP and the subsequent cancer cell proliferation *in vivo*. Consistently, the *in vivo* results demonstrated that the p21-MMP1/MMP9 axis was upregulated by HA-GCX disruption, resulting in the suppression of MP in tumor tissues. Currently, hyaluronidase (HAase) is used to degrade excessive HA of tumor stroma to increase delivery of chemotherapeutic drugs in pancreatic cancer.^54^ Our findings that the administration of HAase in mouse tumor models could delay tumor growth might be attributed to the inhibition of MP in cancer cells. Future comprehensive studies of the HAase application in combination with chemotherapeutic drugs should be pursued, with a particular focus on elucidating the role of MP in this context.

## 5 Conclusions

Our findings demonstrated that HA-GCX thickness was positively correlated with MP activity in cancer cells. Importantly, the impairment of HA-GCX could suppress MP by decreasing cytoskeleton-dependent membrane ruffling. Specifically, we elucidated the role of the p21-MMP1/MMP9 pathway in inhibiting MP, highlighting critical mechanistic insights. Our study may provide a rational basis for developing innovative MP-targeted therapies in cancer intervention.

## Supplementary Material



## Notes

Correlation analysis of TCGA data (Figure S1); dose–response relationship between HAase and MP activity (Figure S2); HA-GCX disruption with 4-MU inhibiting MP (Figure S3); involvement of MP in HA-GCX-mediated cell proliferation (Figure S4); validation of CDKN1A knockdown and knockout efficiency (Figure S5); oligonucleotides (Table S1) (PDF)

## Abbreviations

HA: hyaluronan
HA-GCX: hyaluronan-related glycocalyx
MP: macropinocytosis
HAase: hyaluronidase
CDKN1A/p21: cyclin-dependent kinase inhibitor 1
MMP: matrix metalloproteinase
b-HABP: biotinylated hyaluronan binding protein
TMR-DEX: tetramethylrhodamine-labeled 70 kDa dextran
EIPA: 5-(*N*-ethyl-*N*-isopropyl) amiloride
3D-SIM: three-dimensional structured illumination microscopy
HI: heat-inactivated
4-MU: 4-methylumbelliferone
CytoD: cytochalasin D
CCK-8: Cell Counting Kit-8
GO: gene ontology
GSEA: gene set enrichment analysis
TCGA: The Cancer Genome Atlas
ECM: extracellular matrix
DAPI: 4′,6-diamidino-2-phenylindole
