# Supplementary material for: Impairment of Hyaluronan-Related Glycocalyx Inhibits Macropinocytosis in Cancer Cells via p21 Upregulation
Source: Biomacromolecules. 2025 Sep 3;26(10):6586–99. doi: 10.1021/acs.biomac.5c00812 (PMC12525022; doi:10.1021/acs.biomac.5c00812)
Supplement: Supplementary file 1 [file bm5c00812_si_001.pdf]

Supporting Information

Impairment of Hyaluronan-Related Glycocalyx Inhibits Macropinocytosis in Cancer Cells via p21 Upregulation

Guangxin Luan[1,2†], Qian Guo[1†], Yiwen Liu[1], Guoliang Zhang[1], Yan Du[1], Cuixia Yang[1,2], Hui Wang[1,2], Jiajie Hu[1], Yiqing He[1*] and Feng Gao[1,2*]

[1]Department of Molecular Biology, Shanghai Sixth People's Hospital Affiliated to Shanghai Jiao Tong University School of Medicine, Shanghai, China.

[2]Department of Clinical Laboratory, Shanghai Sixth People's Hospital Affiliated to Shanghai Jiao Tong University School of Medicine, Shanghai, China.

*** Corresponding Authors**

Yiqing He, Department of Molecular Biology, Shanghai Sixth People's Hospital Affiliated to Shanghai Jiao Tong University School of Medicine, Shanghai 200233, P. R. China. E-mail: heyiqing01@163.com.

Feng Gao, Department of Molecular Biology and Clinical Laboratory, Shanghai Sixth People's Hospital Affiliated to Shanghai Jiao Tong University School of Medicine, Shanghai 200233, P. R. China. E-mail: gaofengly@sjtu.edu.cn.

† These Authors Contributed Equally to This Work.

Figure S1.

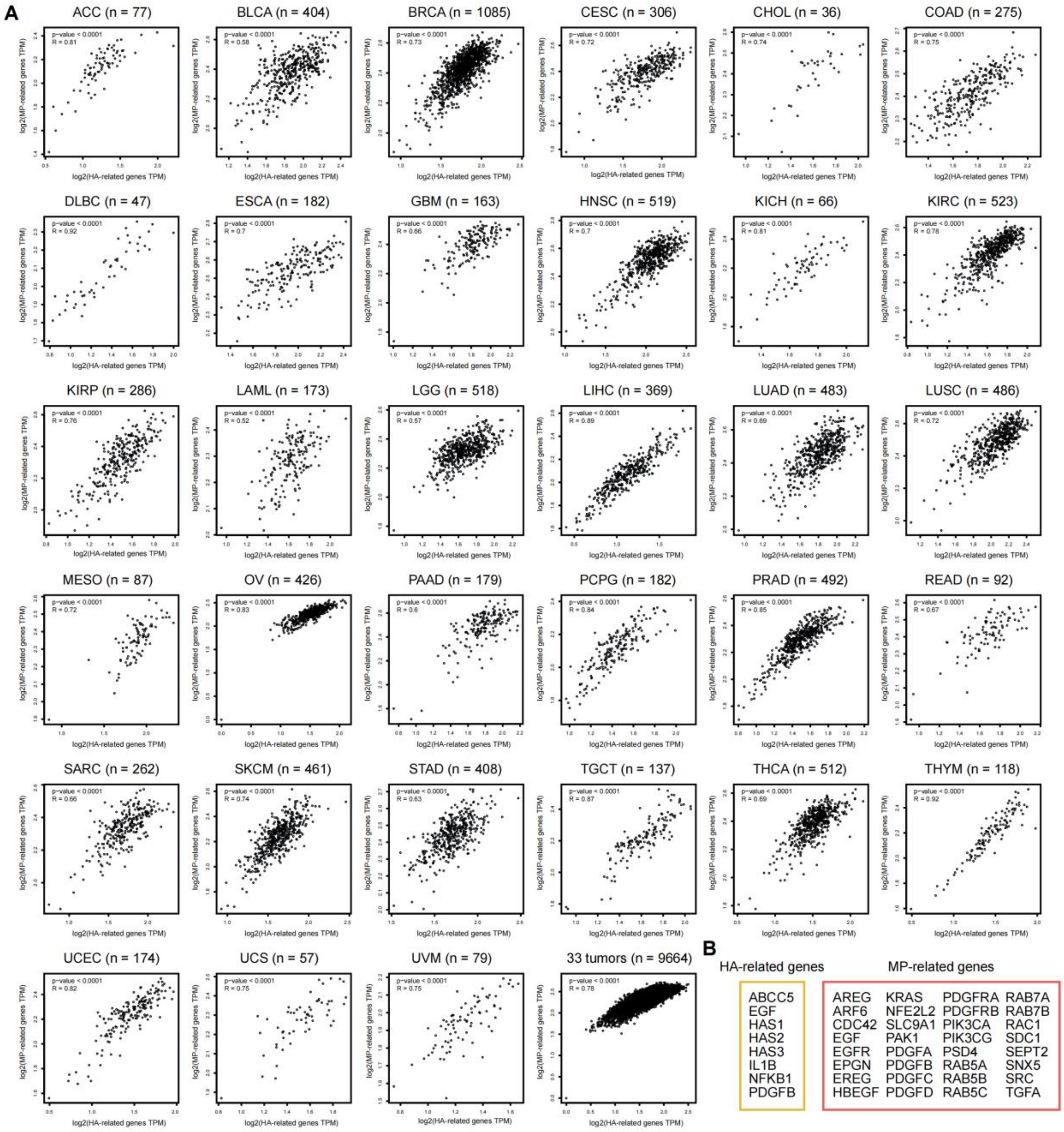

Figure S1. HA synthesis-related signature is positively correlated with MP-related signature across 33 types of human cancers.

(A) Correlation scatter plots between HA synthesis-related signature and MP-related signature across 33 types of human cancers. The *p*-values and Spearman correlation coefficient (R) are indicated. Data are procured from TCGA. **(B)** Lists of HA synthesis-related genes and MP-related genes.

Figure S2.

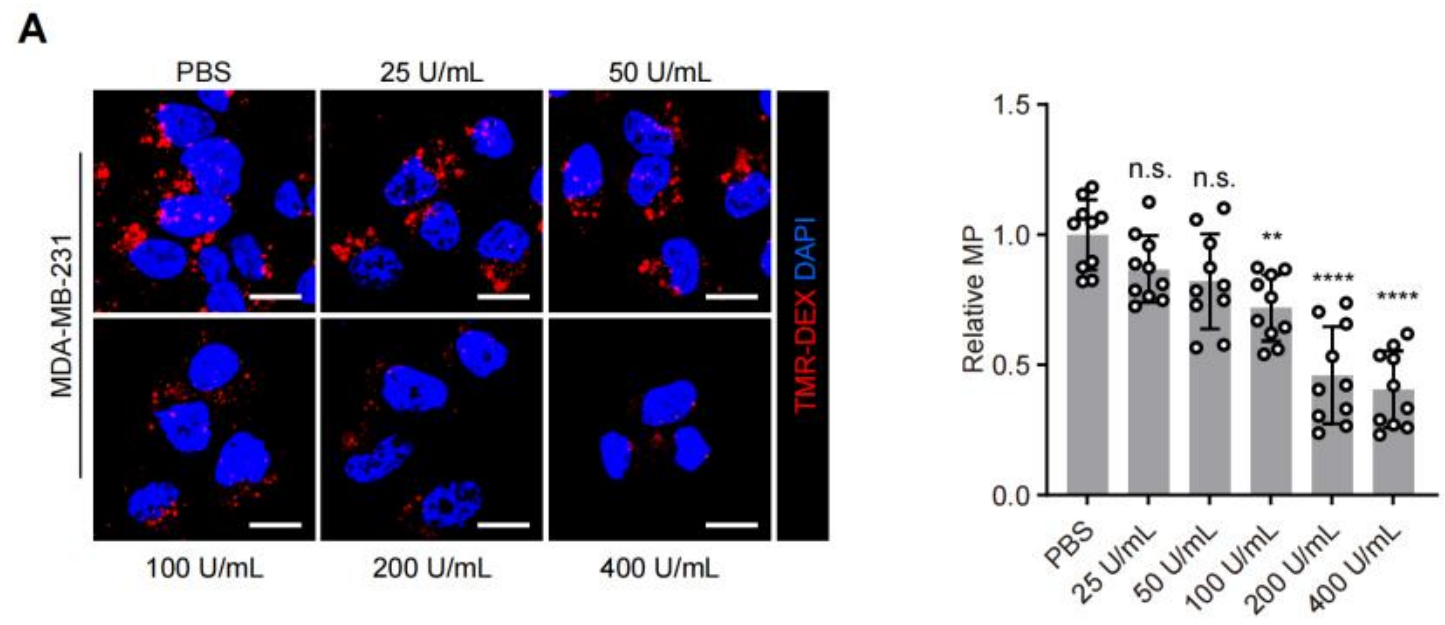

Figure S2. Dose-response relationship between HAase and MP activity in cancer cells.

(A) MP visualization using TMR-DEX (red) in MDA-MB-231 treated with PBS or HAase at indicated doses for 24 h. Scale bars, 10 μm. Quantification of relative MP activity is presented relative to the PBS group. Mean ± SD (n = 10). ** $p < 0.01$, **** $p < 0.0001$.

Figure S3.

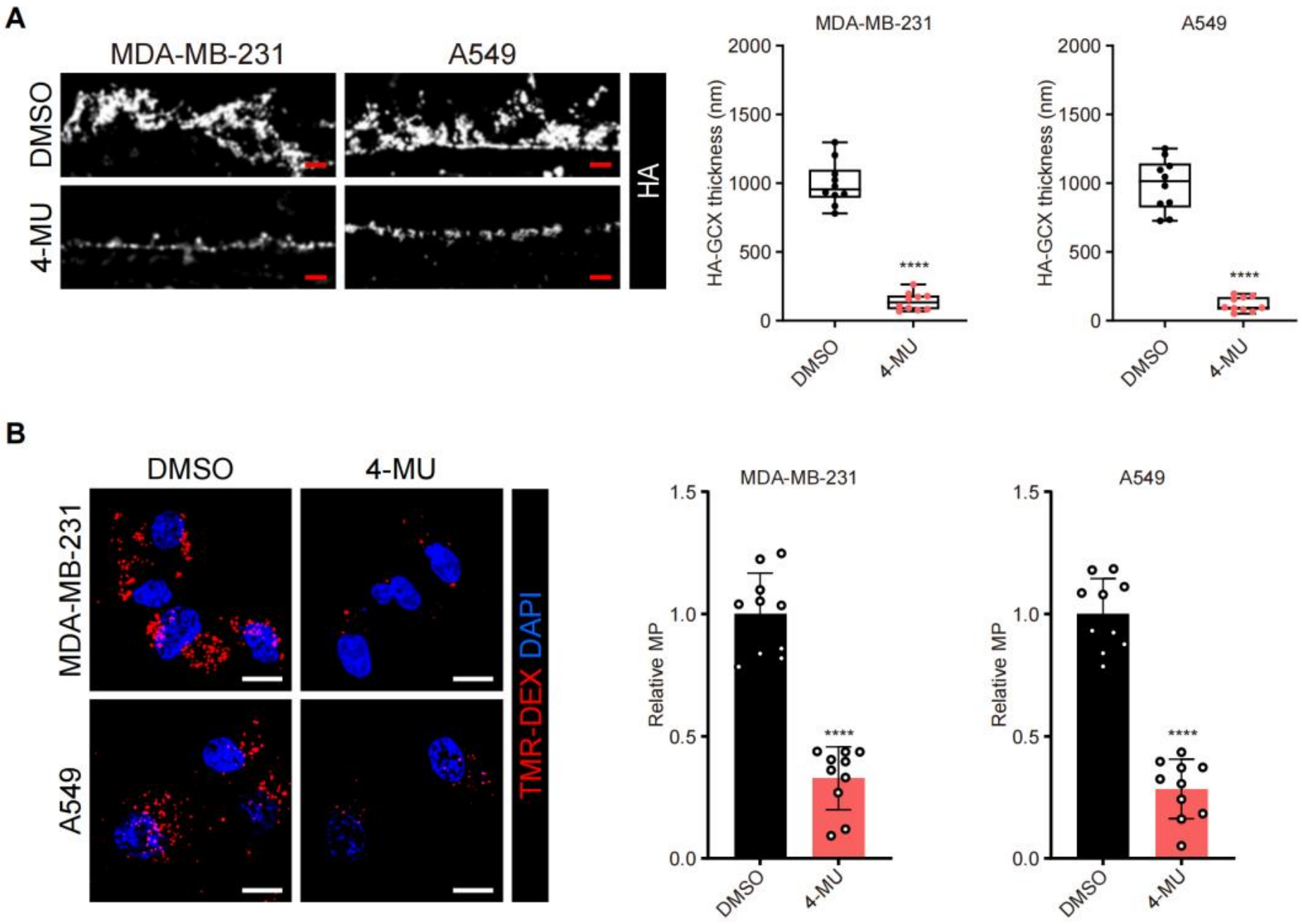

Figure S3. HA-GCX disruption with 4-MU inhibits MP in cancer cells.

(A) Representative 3D-SIM images of HA-GCX (b-HABP, white) in MDA-MB-231 and A549 cells treated with or without 4-MU. Scale bars, 1 μm. Quantification of HA-GCX thickness is performed at 10 cells (10 points per cell) for each group (n = 10 cells). **(B)** MP visualization using TMR-DEX (red) in MDA-MB-231 and A549 cells treated with or without 4-MU. Scale bars, 10 μm. Quantification of relative MP activity is presented relative to the DMSO group. Mean ± SD (n = 10). ****$p < 0.0001$.

Figure S4.

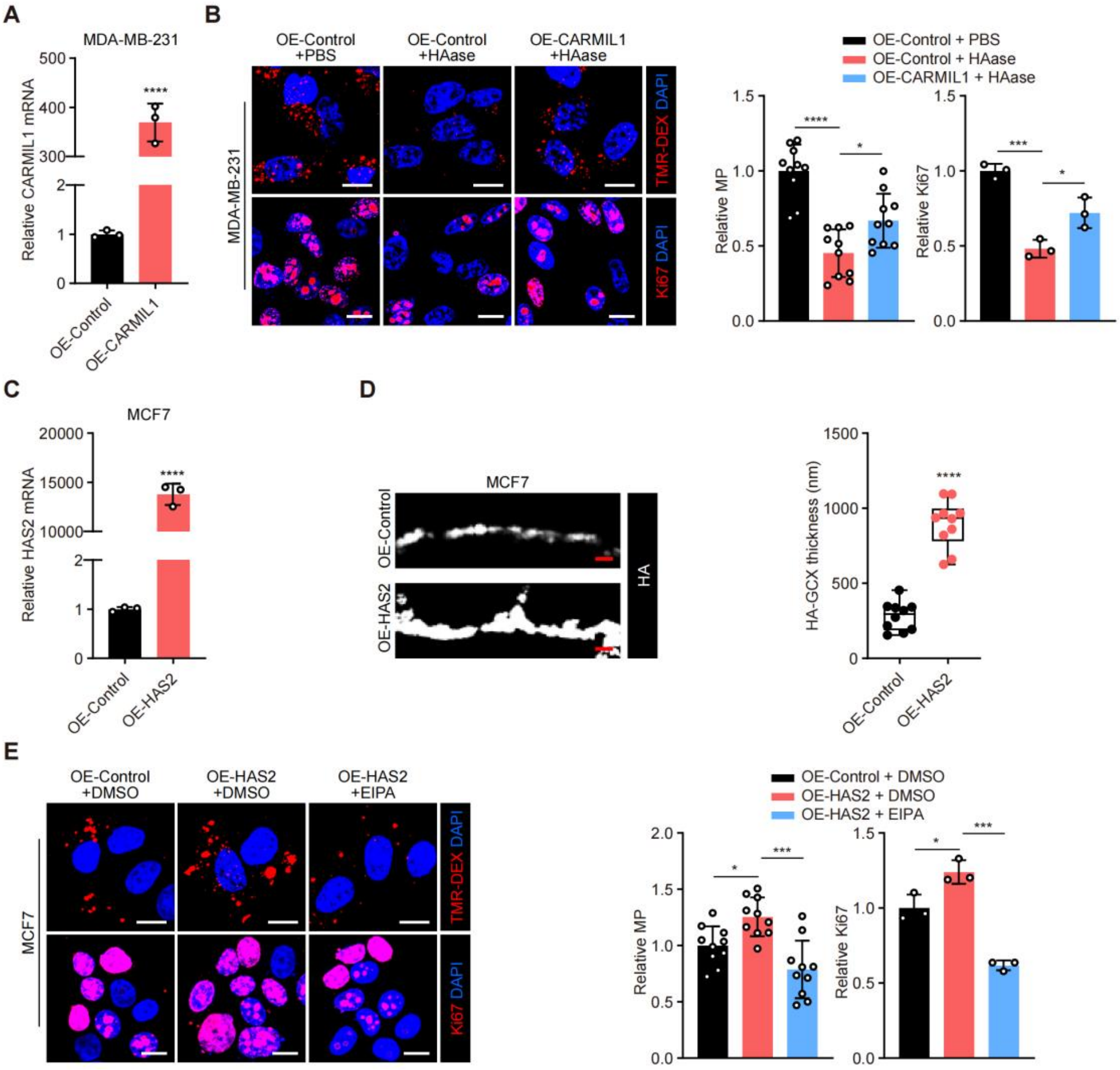

Figure S4. The involvement of MP activity in HA-GCX-mediated cell proliferation.

(A) Analysis of CARMIL1 overexpression efficiency by qPCR in MDA-MB-231 cells. Mean ± SD (n = 3). **(B)** MP visualization using TMR-DEX (red) and representative immunofluorescence staining images of Ki67 (red) in OE-control and OE-CARMIL1 MDA-MB-231 cells treated with or without HAase. Scale bars, 10 µm. Quantification of relative MP activity is presented relative to the OE-control PBS group. Mean ± SD (n = 10). Quantification of relative Ki67-positive cells is presented relative to the OE-control PBS group. Mean ± SD (n = 3). **(C)** Analysis of HAS2 overexpression efficiency by qPCR in MCF7 cells. Mean ± SD (n = 3). **(D)** Representative 3D-SIM images of HA-GCX (b-HABP, white) in OE-control and OE-HAS2 MCF7 cells. Scale bars, 1 µm. Quantification of

HA-GCX thickness is performed at 10 cells (10 points per cell) for each group (n = 10 cells). **(E)** MP visualization using TMR-DEX (red) and representative immunofluorescence staining images of Ki67 (red) in OE-control and OE-HAS2 MCF7 cells treated with or without EIPA. Scale bars, 10 µm. Quantification of relative MP activity is presented relative to the OE-control PBS group. Mean ± SD (n = 10). Quantification of relative Ki67-positive cells is presented relative to the OE-control PBS group. Mean ± SD (n = 3). *$p < 0.05$, ***$p < 0.001$, and ****$p < 0.0001$.

Figure S5.

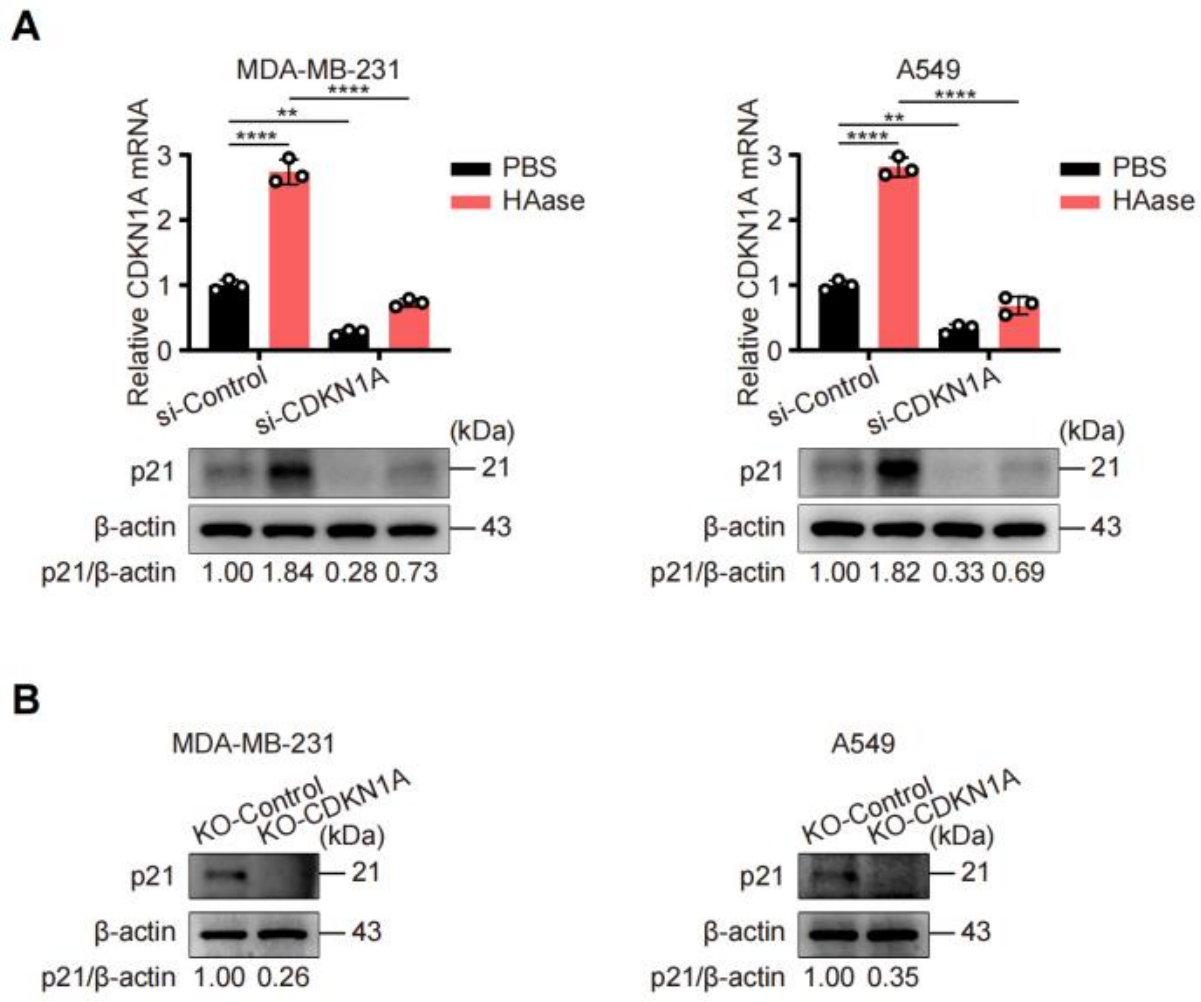

Figure S5. Validation of CDKN1A knockdown and knockout efficiency in cancer cells.

(A) Analysis of CDKN1A/p21 expression by qPCR and Western blotting in si-control and si-CDKN1A MDA-MB-231 and A549 cells treated with or without HAase. Mean ± SD (n = 3). **(B)** Analysis of p21 expression by Western blotting in KO-control and KO-CDKN1A MDA-MB-231 and A549 cells (n = 3). $^{**}p < 0.01$, $^{****}p < 0.0001$.

Table S1. Oligonucleotides

qPCR Primers	Sequence
ACTB Forward	CACCATTGGCAATGAGCGGTTC
ACTB Reverse	AGGTCTTTGCGGATGTCCACGT
ARHGAP18 Forward	GCTGAAGCTCTTCATTCGGGAG
ARHGAP18 Reverse	GGTTCAAAGCCTGTAGTTGCTGC
ASB2 Forward	GGACATCTCCAACAAATCCCGAG
ASB2 Reverse	TTGGTGTCTGCGTTGTGCTGCA
CARMIL1 Forward	CCAAAGTGGACATTAGCGGCAAC
CARMIL1 Reverse	CCTGAAAGCCTTGTGCAGTGATG
CD274 Forward	TGCCGACTACAAGCGAATTACTG
CD274 Reverse	CTGCTTGTCCAGATGACTTCGG
CDKN1A Forward	AGGTGGACCTGGAGACTCTCAG
CDKN1A Reverse	TCCTCTTGGAGAAGATCAGCCG
CTSS Forward	TGGATCACCACTGGCATCTCTG
CTSS Reverse	GCTCCAGGTTGTGAAGCATCAC
ERRFI1 Forward	TGAGGAAGACCTACTGGAGCAG
ERRFI1 Reverse	GTATTAGGCGCTCCTGAGCAGA
FCHSD2 Forward	GCTGCTGTATCAGAACAAAGCCG
FCHSD2 Reverse	TCTGCTTTAGCAGGTCCAACCG
GJB2 Forward	ATCTGGCTCACCGTCCTCTTCA
GJB2 Reverse	TCGTAGCACACGTTCTTGCAGC
HAS2 Forward	GTCATGTACACAGCCTTCAGAGC
HAS2 Reverse	ACAGATGAGGCTGGGTCAAGCA
ITGA2 Forward	TTGCGTGTGGACATCAGTCTGG
ITGA2 Reverse	GCTGGTATTTGTCGGACATCTAG
KLF4 Forward	CATCTCAAGGCACACCTGCGAA
KLF4 Reverse	TCGGTCGCATTTTTGGCACTGG
MMP1 Forward	ATGAAGCAGCCCAGATGTGGAG
MMP1 Reverse	TGGTCCACATCTGCTCTTGGCA
MMP9 Forward	GCCACTACTGTGCCTTTGAGTC
MMP9 Reverse	CCCTCAGAGAATCGCCAGTACT
MTSS1 Forward	TTCAGTGCTCCAGCGGCTACAG
MTSS1 Reverse	GGAATGGTGGAGGACTTGTCGA
STC1 Forward	GCAGGAAGAGTGCTACAGCAAG
STC1 Reverse	CATTCCAGCAGGCTTCGGACAA
**siRNA**	**Sequence**
negative control	siRNA negative control (RiboBio)
CDKN1A	GTGGACAGCGAGCAGCTGA
MMP1	TGAAGATGAAAGGTGGACCAA
MMP9	CCACAACATCACCTATTGGAT
**sgRNA**	**Sequence**
negative control	sgRNA negative control (GeneChem)
CDKN1A	TACCCTTGTGCCTCGCTCAG
